# Supplementary material for: Interplay between epigallocatechin-3-gallate and ionic strength during amyloid aggregation
Source: PeerJ. 2021 Oct 22;9:e12381. doi: 10.7717/peerj.12381 (PMC8544251; doi:10.7717/peerj.12381)
Supplement: Supplemental Information 1 [file peerj-09-12381-s001.pdf]

Interplay between epigallocatechin-3-gallate and ionic strength during amyloid aggregation

Mantas Ziaunys*, Kamile Mikalauskaite, Andrius Sakalauskas, Vytautas Smirnovas

Institute of Biotechnology, Life Sciences Center, Vilnius University, Vilnius, Lithuania

*Corresponding Author:

Mantas Ziaunys

Sauletekio al. 7, Vilnius, LT-10257, Lithuania

Email address: mantas.ziaunys@gmc.vu.lt

Supplementary Information

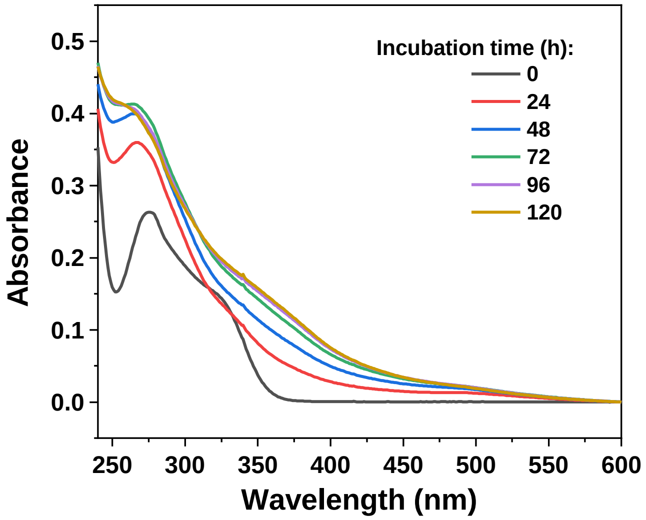

Figure S1. EGCG solution absorbance spectra during incubation. Measurements were taken every 24 hours and each spectrum is the average of three repeats. The measurement procedure is described in the Materials and Methods section.

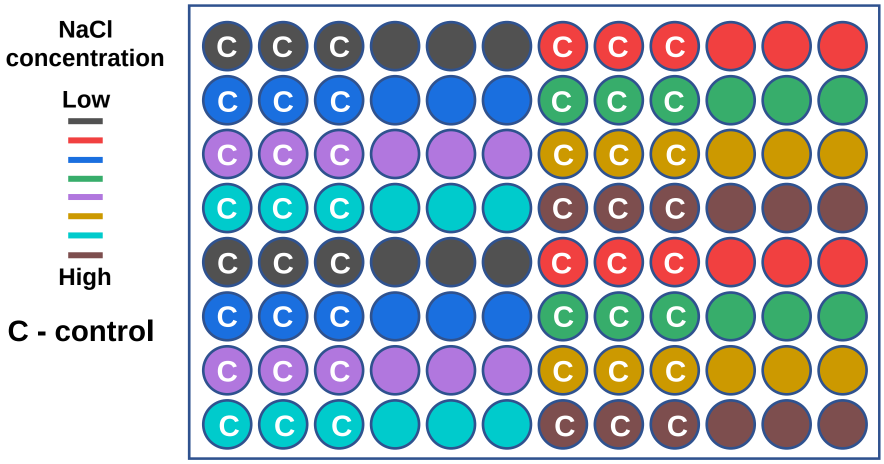

Figure S2. Alternating sample placement in 96-well plates used during all aggregation reactions in order to account for possible uneven temperature distributions. The temperature variation is more extreme vertically (top row to bottom row) rathen than horizontally (column 1 to column 12), which is why the plate is divided into 2 parts. This distribution results in each condition samples being placed an equal average distance from the top and bottom. Each condition samples (colour coded) and their controls (marked with "C") are placed next to each other to minimize variation between them, as they are directly compared against one another.

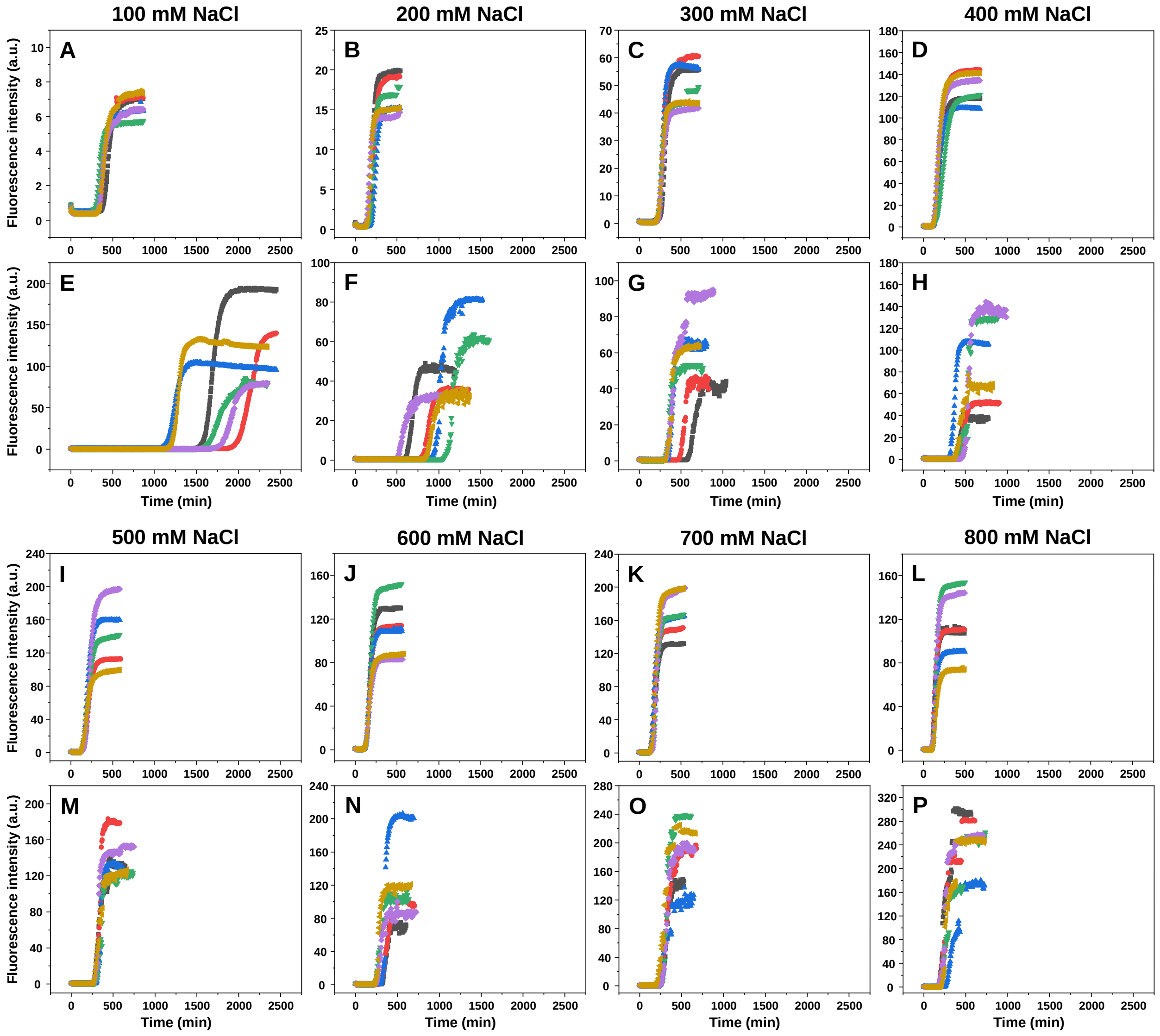

Figure S3. Insulin (200 µM) aggregation kinetic curves in the absence or presence of 25 µM EGCG at 100 mM (A, E), 200 mM (B, F), 300 mM (C, G), 400 mM (D, H), 500 mM (I, M), 600 mM (J, N), 700 mM (K, O) and 800 mM NaCl (L, P). In each case, six repeats are represented by different colours. The displayed kinetic curves were truncated to improve the fitting procedure and are shown as they were fit.

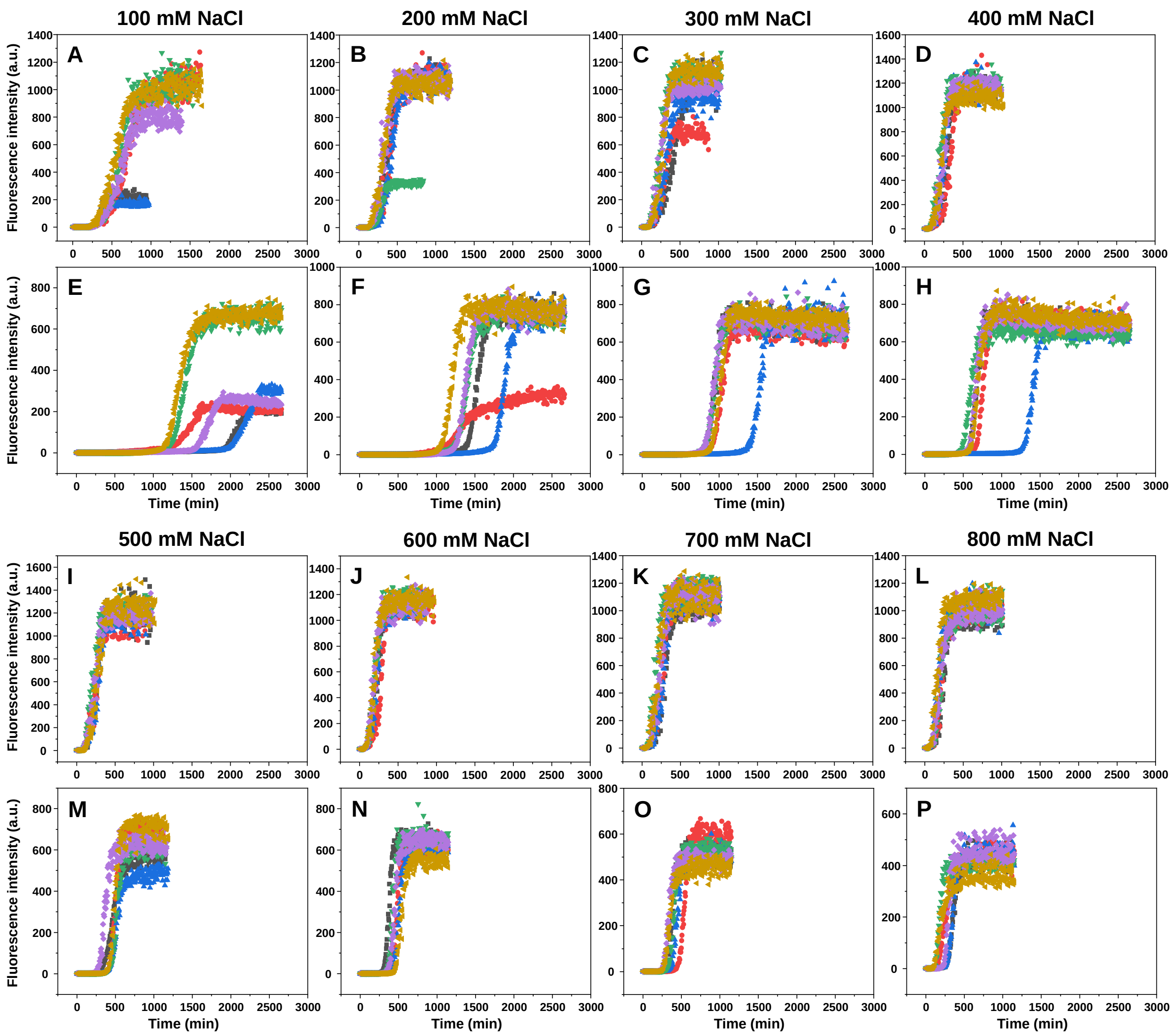

Figure S4. Alpha-synuclein (100 µM) aggregation kinetic curves in the absence or presence of 100 µM EGCG at 100 mM (A, E), 200 mM (B, F), 300 mM (C, G), 400 mM (D, H), 500 mM (I, M), 600 mM (J, N), 700 mM (K, O) and 800 mM NaCl (L, P). In each case, six repeats are represented by different colours. The displayed kinetic curves were truncated to improve the fitting procedure and are shown as they were fit.

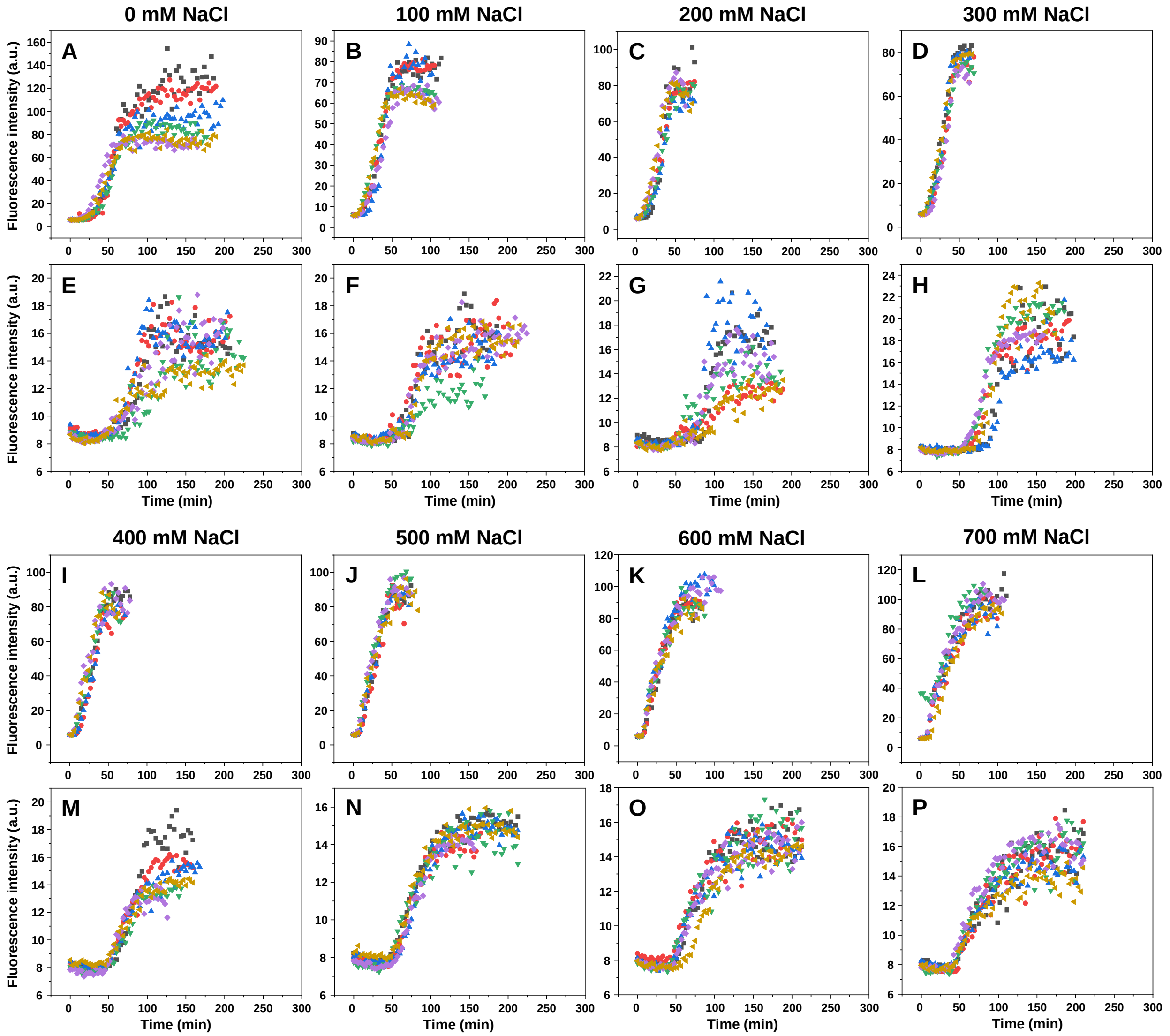

Figure S5. Amyloid-beta (1-42) (2 µM) aggregation kinetic curves in the absence or presence of 25 µM EGCG at 0 mM (A, E), 100 mM (B, F), 200 mM (C, G), 300 mM (D, H), 400 mM (I, M), 500 mM (J, N), 600 mM (K, O) and 700 mM NaCl (L, P). In each case, six repeats are represented by different colours. The displayed kinetic curves were truncated to improve the fitting procedure and are shown as they were fit.

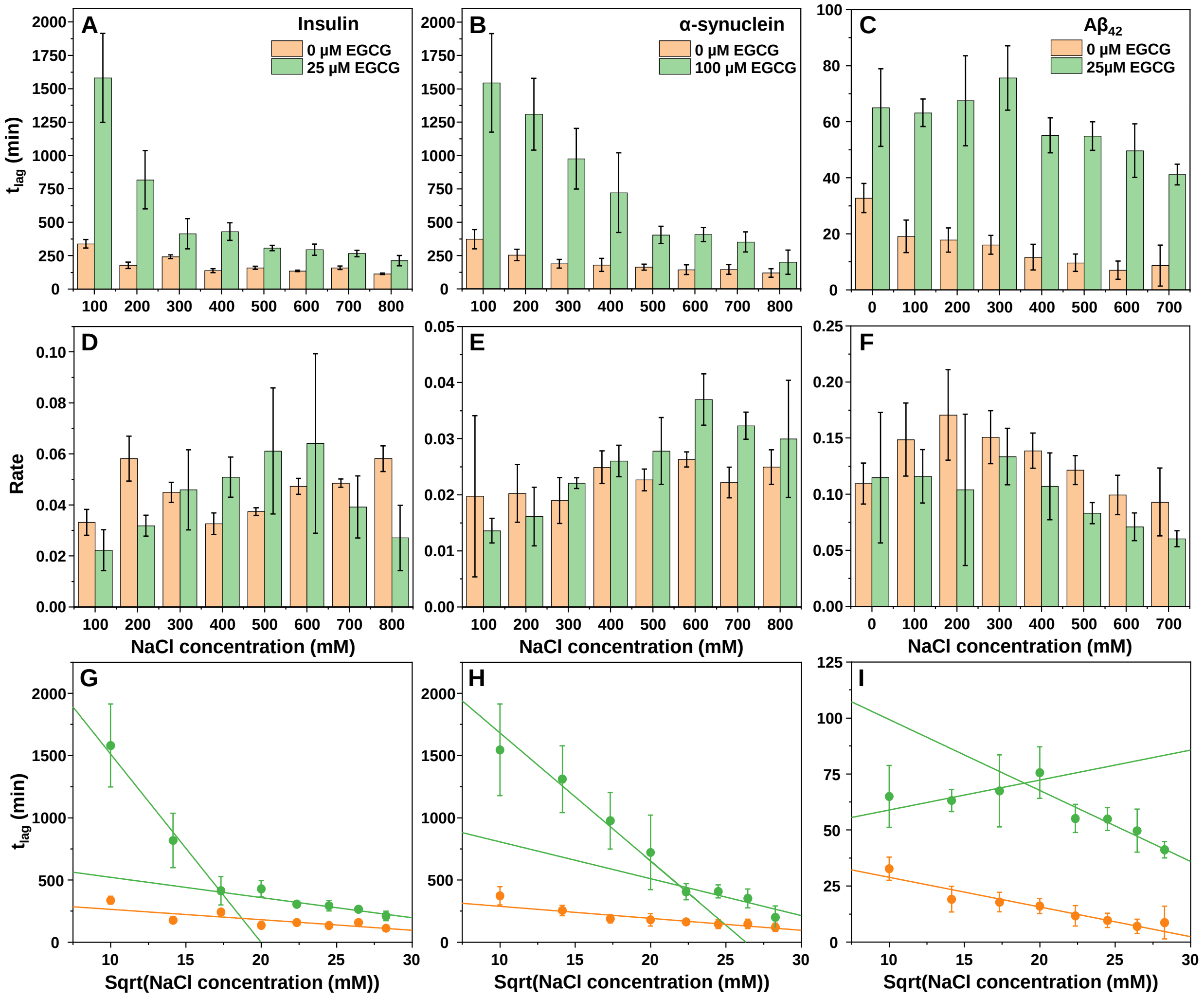

Figure S6. Insulin (A, D, G), alpha-synuclein (B, E, H) and amyloid-beta (1-42) (C, F, I) lag time, aggregation rate and lag time dependence on the square root of NaCl concentration respectively. Lag time and aggregation rate were determined as described in the Materials and Methods section.

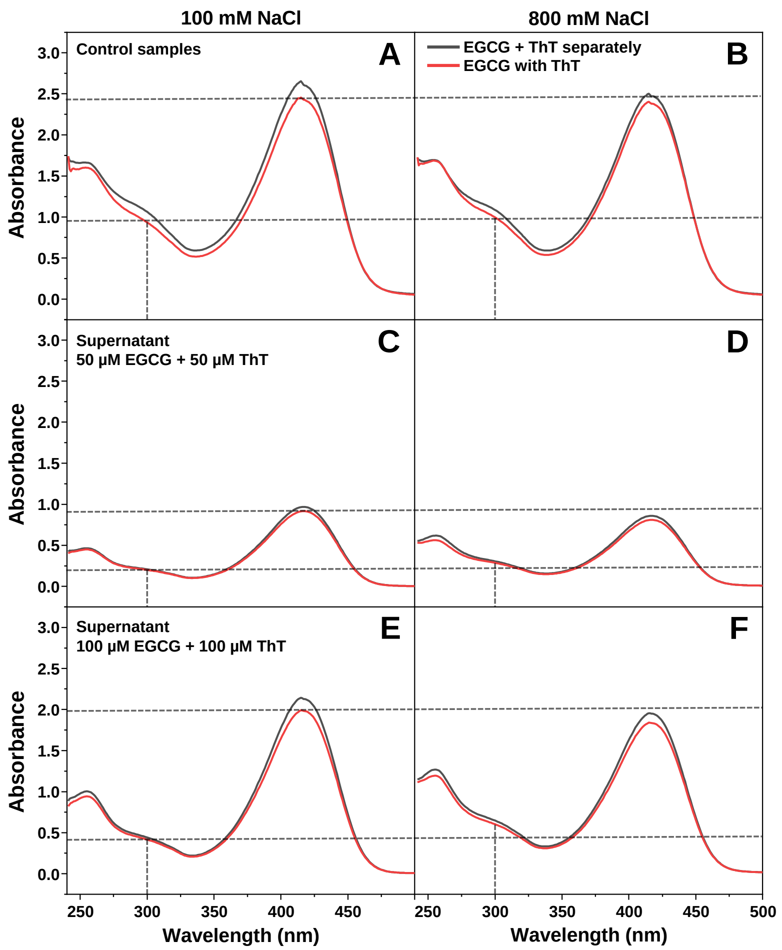

Figure S7. Absorbance spectra of ThT and EGCG when they are separate or together in different ionic strength solutions. 100 µM ThT and 100 µM EGCG absorbance spectra when they are separate or together in 20% acetic acid solutions, containing 100 mM NaCl (A) or 800 mM NaCl (B). Absorbance spectra of supernatants, when ThT and EGCG were combined with insulin fibrils and centrifugated as described in the Materials and Methods section (50 µM ThT and 50 µM EGCG in 20% acetic acid solutions, containing 100 µM insulin fibrils and 100 mM NaCl (C) or 800 mM NaCl (D); 100 µM ThT and 100 µM EGCG in 20% acetic acid solutions, containing 100 µM insulin fibrils and 100 mM NaCl (E) or 800 mM NaCl (F)

Amyloid-beta purification protocol

Aβ_{42} was purified similarly to previously described methods [1–3]. The expression vector for Aβ_{42} (pet3a_AB_1-42_WT) was transformed into Ca2+ competent E. coli cells by the heat shock and covered evenly on LB agar plates containing ampicillin (100 µg/mL). The LB agar plates where incubated for 14 h at 37 °C. The cultures were then prepared from single colonies and grown in LB medium with ampicillin (100 µg/mL) until the medium's OD_{850} reached 0.7. Afterwards, 1 mL of the culture was transferred to 400 mL of auto-inductive ZYM-5052 medium 40 containing ampicillin (100 µg/mL) and grown for 16 h. The cell pellet was collected by centrifuging the cell suspension at 5 000 g at 4°C for 20 minutes. To remove soluble proteins, the pellet was washed 3 times by repeating the following procedure. First, the cell pellet was homogenized with Potter-Elvehjem homogenizer in 150 mL of 20 mM Tris/HCL pH 8.0 buffer containing 1 mM EDTA. Then, it was sonicated for 10 minutes on ice using Sonopuls 3100 (Bandelin) ultrasonic homogenizer equipped with a VS70/T tip (70% power, 30 s sonication / 30 s rest intervals). The cell homogenate was centrifuged at 18 000 g at 4°C for 20 minutes. Last, the supernatant was removed and the cell pellet was washed again. After removing soluble proteins, the cell pellet was resuspended in 50 mL of 20 mM Tris/HCL pH 8.0 buffer solution containing 8 M urea and 1 mM EDTA, homogenized and centrifuged as in the previous steps. The solubilized inclusion body solution was diluted with 150 mL of 20 mM Tris/HCL pH 8.0 buffer containing 1 mM EDTA and mixed with 50 mL DEAE-sepharose equilibrated with the same buffer solution. The mixture was agitated at 80 rpm for 30 minutes at 4 °C. The separation procedure was performed using a Buchner funnel with Fisherbrand glass microfiber paper on a vacuum glass bottle. The DEAE-sepharose with bound proteins was washed with a 20 mM Tris/HCL pH 8.0 buffer containing 1 mM EDTA. The nonspecifically bound proteins were washed with 50 mL of buffer solution with 25 mM NaCl followed with the target protein elution using four aliquouts of buffer solution containing 150 mM NaCl. The collected target peptide fractions were mixed together (200 mL) flash-frozen, lyophilized and stored at -20 ◦C.

After lyophilization, the Aβ42 peptide powder was dissolved in a 20 mM sodium phosphate buffer solution containing 6 M GuHCl (pH 8.0). Then the sample was loaded on a Tricon 10/300 column (packed with Superdex 75 gelfiltration resin) and eluted at 1 mL/min using a 20 mM sodium phosphate buffer solution (pH 8.0), containing 0.2 mM EDTA and 0.02% NaN_3.

References

[1] D.M. Walsh, E. Thulin, A.M. Minogue, N. Gustavsson, E. Pang, D.B. Teplow, S. Linse, A facile method for expression and purification of the Alzheimer's disease-associated amyloid β-peptide, FEBS J. 276 (2009) 1266–1281. https://doi.org/10.1111/j.1742-4658.2008.06862.x.

[2] E. Hellstrand, B. Boland, D.M. Walsh, S. Linse, Amyloid β-protein aggregation produces highly reproducible kinetic data and occurs by a two-phase process, ACS Chem. Neurosci. 1 (2010) 13–18. https://doi.org/10.1021/cn900015v.

[3] H. Vignaud, C. Bobo, I. Lascu, K.M. Sörgjerd, T. Zako, M. Maeda, B. Salin, S. Lecomte, C. Cullin, A structure-toxicity study of Aß42 reveals a new anti-parallel aggregation pathway, PLoS One. 8 (2013) 1–13. https://doi.org/10.1371/journal.pone.0080262.
